# Efficacy of a Student-Led, Community-Based, Multifactorial Fall Prevention Program: Stay in Balance

**DOI:** 10.3389/fpubh.2017.00030

**Published:** 2017-02-27

**Authors:** Cheryl A. Der Ananian, Melanie Mitros, Matthew Paul Buman

**Affiliations:** ^1^School of Nutrition and Health Promotion, Arizona State University, Phoenix, AZ, USA; ^2^Vitalyst Health Foundation, Phoenix, AZ, USA

**Keywords:** exercise or physical activity, aging, fall prevention, balance, physical function, health promotion

## Abstract

**Background:**

Falls are a major public health concern in older adults. Recent fall prevention guidelines recommend the use of multifactorial fall prevention programs (FPPs) that include exercise for community-dwelling older adults; however, the availability of sustainable, community-based FPPs is limited.

**Methods:**

We conducted a 24-week quasi-experimental study to evaluate the efficacy of a community-based, multifactorial FPP [Stay in Balance (SIB)] on dynamic and functional balance and muscular strength. The SIB program was delivered by allied health students and included a health education program focused on fall risk factors and a progressive exercise program emphasizing lower-extremity strength and balance. All participants initially received the 12-week SIB program, and participants were non-randomly assigned at baseline to either continue the SIB exercise program at home or as a center-based program for an additional 12 weeks. Adults aged 60 and older (*n* = 69) who were at-risk of falling (fall history or 2+ fall risk factors) were recruited to participate. Mixed effects repeated measures using Statistical Application Software Proc Mixed were used to examine group, time, and group-by-time effects on dynamic balance (8-Foot Up and Go), functional balance (Berg Balance Scale), and muscular strength (30 s chair stands and 30 s arm curls). Non-normally distributed outcome variables were log-transformed.

**Results:**

After adjusting for age, gender, and body mass index, 8-Foot Up and Go scores, improved significantly over time [*F*_(2,173)_ = 8.92, *p* = 0.0; T0 − T2 diff = 1.2 (1.0)]. Berg Balance Scores [*F*_(2,173)_ = 29.0, *p* < 0.0001; T0 − T2 diff = 4.96 (0.72)], chair stands [*F*_(2,171)_ = 10.17, *p* < 0.0001; T0 − T2 diff = 3.1 (0.7)], and arm curls [*F*_(2,171)_ = 12.7, *p* < 0.02; T0 − T2 diff = 2.7 (0.6)] also all improved significantly over time. There were no significant group-by-time effects observed for any of the outcomes.

**Conclusion:**

The SIB program improved dynamic and functional balance and muscular strength in older adults at-risk for falling. Our findings indicate continuing home-based strength and balance exercises at home after completion of a center-based FPP program may be an effective and feasible way to maintain improvements in balance and strength parameters.

## Introduction

Falls are a significant public health concern for older adults. Fall-induced injuries are one of the most common causes of restricted activity, disability, and death in elderly populations (1). In 2014, nearly 29% of adults over the age of 65 reported a fall, resulting in 7 million injuries (2). The percentage of individuals who experience a fall and the percentage of individuals who report a fall injury both increase with age. The consequences of falls vary from relatively minor to severe. Falls are the leading cause of injury-related deaths and non-fatal injuries in older adults (3). In 2014, 2.8 million adults over the age of 65 were treated in emergency departments for fall-related injuries, and approximately 800,000 were hospitalized for the severity of their injuries (2). Among adults over the age of 65, falls account for nearly 55% of all deaths from unintentional injuries, and falls-related mortality has steadily increased from 2000 to 2013. The age-adjusted death rate from falls-related injuries has nearly doubled from 29.6 per 100,000 in 2000 to 56.7 per 100,000 in 2013 (4).

Falls-related injuries are one of the most expensive medical conditions, and the costs associated with falls are expected to rise as the US population ages. In 2015, direct medical costs for fatal and non-fatal injuries were $637.5 million and 31.3 billion, respectively. Importantly, costs for fatal and non-fatal fall injuries increased 21 million and 1 billion dollars, respectively, from 2014 to 2015 (5). Costs and incidence of falls increased with age. Similarly, women had a greater incidence of falls and higher costs. Direct costs do not account for the long-term effects of these injuries such as disability, dependence on others, lost time from work and household duties, and reduced quality of life, all of which are important considerations for falls-related outcomes.

Recent national guidelines highlight the importance of exercise for fall prevention. Specifically, the US Preventive Services Task Force stated that participation in physical activity and vitamin D supplementation were the only two individual strategies with sufficient evidence to recommend for fall prevention (6). Participation in regular physical activity and, more specifically, exercises targeting lower extremity strength and balance has been shown to reduce the risk of falling and improve balance outcomes (7–11). Similarly, the Centers for Disease Control and Prevention released a framework for community-based fall prevention programs (FPPs) (12, 13). This framework recommends multicomponent programs that target multiple risk factors including physical activity, vision, medication use, and environmental changes.

While physical activity is highlighted as a critical component of multifactorial FPPs, a major challenge for delivering community-based FPPs targeting exercise is developing cost-effective, sustainable programs. For example, Sherrington and colleagues (10, 11) concluded exercise programs need to have a minimum of 50 contact hours over 24 weeks and include a progressive balance and resistance training program to be effective. Their research suggested exercise intervention programs may reduce the risk of falling by up to 17% if properly implemented. However, this extended program length may not be feasible for community-based settings due to space and time restrictions. Similarly, not all settings have trained exercise physiologists on staff, and developing a sustainable model for delivering an FPP with qualified staff can be problematic. To address the feasibility and sustainability of community-based exercise programs, the input and collaboration of community-based organizations is necessary during the development process. Community-based FPPs need to align with the needs and resources of community organizations.

To address these potential issues with sustainability, Stay in Balance (SIB) was developed in conjunction with community partners as a student-led, community-based, multifactorial FPP using allied health-care students to deliver the program. The purpose of this study was to evaluate the efficacy of the SIB Program on balance and physical function outcomes. Additionally, we evaluated the impact of two different models for ongoing sustainability—12 weeks of home-based versus 12 weeks of center-based exercise—after completion of the SIB Program on balance and physical function outcomes. We hypothesized that all participants would have significant improvements in measures of balance and physical function after completing the 12-week, multifactorial SIB Program and there would be no differences in these outcomes between the follow-up home-based or center-based programs at 24 weeks.

## Materials and Methods

### Study Design

This study assessed the efficacy of a 12-week, multicomponent FPP, SIB, plus either 12 weeks of a home-based or center-based exercise program on balance and physical function in older adults using a quasi-experimental design with multiple posttests. *All participants* received the multifactorial, 12-week SIB Program. At the end of the 12-week SIB Program, participants were assigned to either continue the exercise portion of the SIB Program at home or as part of a center-based program for 12 weeks. Allocation to continue the exercise portion of the SIB Program at home or within a center was determined at the beginning of the study based on site preference and availability. Participants in sites that could not commit to hosting the program for 24 weeks were allocated to the home-based exercise program; participants in sites that could host the program for 24 weeks were allocated to the center-based exercise program. Outcome variables were measured at the beginning of the program, at the end of the 12-week multifactorial SIB Program and again at 24 weeks. At each time point, participants completed the balance and physical function assessments.

### Informed Consent

This study was approved by the Arizona State University Institutional Review Board and was carried out in accordance with the policies and guidelines set forth by the Office of Research Integrity and Assurance at Arizona State University. All participants provided informed consent in accordance with the Declaration of Helsinki prior to participating in the study.

### Recruitment

To facilitate recruitment and identify potential locations to deliver the SIB Program, a partnership was established with the Greater Valley Area Health Education Center (GVAHEC) Empowerment Systems, Inc. (EmSys). Sites to deliver the program were recruited from the greater Phoenix metropolitan area in Maricopa County with the assistance of allied health-care student interns at GVAHEC/EmSys. Community programs throughout the Phoenix metropolitan area were contacted by the interns to determine their interest in receiving this FPP. From these interactions, nine locations were approached as potential sites for the SIB Program. Two locations did not feel that the SIB Program would be appropriate for their community members, three did not have the facilities/scheduling available to host the SIB Program, and one location would not approve an outside organization providing programming. Therefore, three sites were selected based on the location’s interest and availability as well as the number of older adults interested at these locations. One site offered two instances of the program with no overlap of program participants between the two instances of the program.

Community-dwelling older adults were recruited from the three identified sites. To maximize contact within the community at each site, methods of recruitment varied according to site needs. All recruitment strategies were facilitated by employees from the respective sites. Strategies included posted flyers, information sessions, interacting with potential participants during lunch, and electronic advertising in community newsletters and/or community websites.

### Inclusion/Exclusion Criteria

Inclusion/exclusion criteria were chosen to produce a group of older adults who had an elevated risk for falls. The inclusion criteria included: 60–100 years of age; a self-report of a fall in the past 12 months or two or more of the following criteria: age >80 years, self-reported osteoarthritis of lower extremity, taking four or more medications, self-report of fear of falling or concern about falling, or physically inactive [self-report of a Rapid Assessment of Physical Activity (RAPA) score of less than 6 (14)]. Participants were also required to self-report the ability walk one city block with or without an assistive device, to be able to follow directions and complete questionnaires in English and provide informed consent to participate. The exclusion criteria were plans to leave the Phoenix metropolitan area during the program; significant cognitive impairment on the Short Portable Mental Status Questionnaire (SPMSQ) as evidenced by answering five or more questions incorrectly (15, 16); a score of 10 or higher on the Centers for Epidemiological Studies—Depression 10 (CESD-10) (17) suggesting evidence of depression; self-report of uncontrolled chronic illness including heart disease, hypertension, diabetes, or angina; knee or hip replacement within the past 12 months; and failure to meet requirements of the Revised Physical Activity Readiness Questionnaire (R PAR-Q) (18) or to obtain permission from their healthcare provider if they did not meet the requirements of the R PAR-Q. Individuals who expressed interest in the SIB Program were initially screened for eligibility in person or by telephone. The initial screening consisted of sociodemographic, general health history (including cardiovascular disease, fall and lower extremity joint replacement history), and physical activity questions. Older adults who met the initial eligibility criteria were asked to attend an in-person meeting to complete further screening for cognitive impairment, depression, and contraindications to exercise (SPMSQ, R PAR-Q, and CESD-10) after informed consent was obtained.

### Theoretical Framework

The SIB Program was grounded in the Social Cognitive Theory (SCT) (19). The SCT describes learning in terms of the reciprocal relationship between behavior, environmental factors, and personal factors. According to SCT, the learner acquires knowledge as his or her environment converges with personal characteristics and personal experience. New experiences are evaluated *via* the past; prior experiences help to subsequently guide and inform the older adult as to how the present should be interpreted and what action should be taken. Using the SCT to design FPPs may help older adults identify the important influences on their sense of control over the consequences of aging. These influences include an individual’s judgment regarding ability to perform a specific behavior (self-efficacy), the person’s beliefs about the effectiveness of his or her own actions (outcome expectations), and the explanations the person gives for outcomes (attributions). Self-efficacy is a person’s confidence in their ability to perform a specific behavior (19). Self-efficacy is situational and is a person’s confidence in their ability to do the said task, such as being physically active and completing daily activities without falling.

The SIB Program was designed to improve the participants’ falls and physical activity self-efficacy. One of the leading causes of activity restriction in older adults is fear of falling, and this is a major focus of this intervention (20–23). By observing and interacting with their peers in a group setting such as the SIB Program, older adults can preserve or enhance their sense of self-efficacy while changing their abilities. Similar to other physical activity interventions, attitudes and beliefs are addressed before behavior change strategies are implemented (24). The educational component of the SIB Program used goal setting, self-monitoring, stimulus control, and reinforcement strategies throughout to increase self-efficacy.

### Intervention Description

The SIB Program was developed as a community partnership between Arizona State University, the GVAHEC/EmSys, and a local senior transitional retirement community center. The SIB Program was premised on the 2008 CDC Compendium of FPPs, which recommended the use of multicomponent programs to address falls (12). Consistent with recommendations set forth in this compendium, the SIB Program focused on known, modifiable risk factors for falls including education about falls risk factors, polypharmacy, vision, home modifications, diet and bone health (vitamin D and calcium), and exercise. The intervention was delivered by allied health-care students, primarily Master’s students studying exercise science and/or health promotion. All students were trained by a doctoral student prior to implementing the program. The training was approximately 4 h in length and included hands-on demonstrations of the exercise program and the health education program by research staff. The students were required to role play during the training and to successfully demonstrate the program back to the research staff during the training. Two Master’s level students and a doctorate student were the primary class leaders with assistance from Bachelor’s level students.

The SIB Program consisted of twice weekly 90 min sessions for a duration of 12 weeks. The first 55–60 min of the session was exercise while the remaining 30 min was health education regarding falls risk factors and strategies for maintaining physical activity. The exercise routine included a 5-min warm-up, 25 min of individualized progressive resistance training with exercise bands and ankle weights primarily focused on lower extremity strength, 15 min of progressive balance exercises, and 10 min of cooldown and flexibility exercises. Participants were asked to complete the exercises on their own at home one time per week during the SIB Program. The health education program was designed to facilitate discussion about falls risk factors and physical activity. Topics discussed included education about falls risk factors, vision assessment, polypharmacy and medication management, home modifications, calcium and vitamin D supplementation, physical activity and falls, and strategies to promote participation in physical activity. All lessons and exercise classes were taught by allied health-care students in an interactive manner to facilitate group participation and retention of information.

All participants were provided with an exercise manual that included safety tips, intervention exercise expectations, detailed descriptions and pictures of the exercises, and an exercise log to facilitate the once weekly home exercise sessions. The exercise manual was taken home on the first day of class to be used as a resource for home exercise days. All participants were also provided with a health education manual that included health information and worksheets on the respective topics. This health education manual was used in class and was given to the participants to keep on the last day of class. Additionally, they received a video of the exercise program on the last day of class to encourage continued physical activity at home.

### Measures

#### Sociodemographic Characteristics and Health History

Participants were asked to report their sociodemographic characteristics including age, gender, race/ethnicity, education level, income level, marital status, and whether or not they lived alone. Participants were also asked to report the presence of chronic illness, medication use, the need for assistive devices, and fall history.

#### Anthropometric Measures

Participants were weighed to the nearest 0.1 kg in light indoor clothing, without shoes when feasible, and pockets emptied. Height was measured to the nearest 0.2 cm with a portable stadiometer without shoes when feasible. Waist circumference was assessed using a Gulick tape measure with the participant standing, at the midpoint between the inferior aspect of the last rib and the superior aspect of the iliac crest, over light indoor clothing to the nearest 0.5 cm. Waist circumference was measured twice and averaged.

#### Physical Activity

At baseline, physical activity was assessed with the RAPA questionnaire. This 9-item questionnaire assesses participation in sedentary through vigorous physical activity, as well as participation in strength training and flexibility exercises (14). The total score of the first 7 items is from 1 to 7 points based on the participant’s response (yes or no). Physical activity is then categorized into one of five levels of physical activity: 1 = sedentary, 2 = underactive, 3 = regular underactive (light activities), 4 = regular underactive, and 5 = regular active. Responses to the strength training and flexibility items are scored separately, with strength training = 1, flexibility = 2, or both = 3. When compared to the Community Healthy Activities Model Program for Seniors physical activity questionnaire, the RAPA is moderately correlated to the self-reported moderate caloric expenditure (*r* = 0.54); it also showed good sensitivity (81%), positive predictive value (77%), and negative predictive value (75%) (14).

#### Fear of Falling

The Fall Efficacy Scale International (FES-I) is a 16-item self-report questionnaire used to asses concern about falls in older adults (25). It is scored on a 4-point scale (1 = not at all concerned to 4 = very concerned). The 16-item scores are summed to get a final score. Cut-points for low and high concern were recently established as FES-I score of 16–22 for low and 23–64 for high concern (26); therefore, a score of greater than or equal to 23 would indicate a high concern for falling. The FES-I has been found to be reliable and valid in multiple cultures and languages (27).

#### Dynamic Balance

The 8-Foot Up and Go (28) was used to assess dynamic balance. Participants were asked to rise from a seated position, walk 8 ft, turn about a cone, and return to a seated position, and the time it took to complete this task was recorded to the nearest 1/100th of a second. The participants completed one practice trial and two test trials; the average score of the two trials was scored. The 8-Foot Up and Go is a valid and reliable measure (28) and has predictive ability for declines in physical function (29).

#### Functional Balance

The Berg Balance Scale (BBS) was used to assess functional balance. The BBS is comprised of 14 items that challenge one’s balance. Each item is scored from 0 to 4 (30–32) with a maximum possible total score of 56. BBS scores are moderately to highly correlated with numerous functional assessments (e.g., gait speed, postural sway, and TUG) (33) and the instrument has high inter- [intraclass correlation coefficient (ICC) = 0.98] and intra-rater reliability (ICC = 0.98) (30). Scores of less than 45 have been shown to be predictive of multiple falls (34, 35). Donoghue et al. (36) determined the minimal detectable change score necessary on the BBS for improvement in falls outcomes. Specifically, they determined the minimal detectable change score varied by starting point: a change of 4 points or more is necessary for BBS scores between 45 and 56, 5 or more points for scores between 35 and 44, 7 or more points from 25 to 34, and 5 points for those scoring between 0 and 24 (36).

#### Physical Function

The Senior Fitness Test by Rikli and Jones (28) was used to assess aspects of physical function. Muscular strength was measured by the 30-s repeated chair stand (28, 37) and the 30-s repeated arm curl (28). If the participant was unable to stand without using their arms for assistance, they were permitted to complete the 30-s chair task while using their arms but they received a score of zero. The 30-s chair stand has been shown to be a reliable (ICC = 0.84–0.92) and valid measure of lower extremity strength (*r* = 0.71–0.78) in laboratory settings (38). For the 30-s arm curl test, males, and females used an 8 and 5-lb dumbbell, respectively, and were instructed to perform a bicep curl as many times as they could in 30 s. If they were unable to curl the specified weight, they were allowed to complete the task without the dumbbell, but they received a 0 score. The repeated arm curls are a valid (*r* = 0.78) and reliable (*r* = 0.81) (28) measure of arm strength.

### Statistical Analysis

All statistical analyses were performed using the Statistical Application Software (SAS) software (version 9.1.3, SAS Institute Inc., Cary, NC, USA). Statistical significance for this study was set at the *p* < 0.05 level. Kolmogorov–Smirnov tests for normality were used to examine all outcome variables (8-Foot Up and Go, Berg Balance, 30-s chair stands, and the 30-s arm curls). Descriptive statistics were computed for demographics and physical assessment data. To determine if differences existed between program completers and non-completers, independent *t*-tests (for normally distributed data), Wilcoxon Rank Sum (for non-normally distributed data) tests, and chi-squared tests (for categorical data) were conducted. Analyses of outcome measures were conducted on program completers only. To examine changes over time in outcome variables, linear growth model analyses were conducted using SAS Proc Mixed. The analyses were conducted in a hierarchical fashion using Restricted Maximum Likelihood model and “autoregressive heterogeneous 1” covariance error structure. The effect of time was evaluated using linear and quadratic trajectories and time × group effects examined the between-group differences in the trajectories during the sustainability phase of the program. Linear growth model analyses controlled for age, gender, and BMI at baseline and clustered on program/site location. Cohen’s *d* effects size estimates were calculated to assess the magnitude of effects and were interpreted using standard conventions for small (*d* = 0.2), medium (*d* = 0.5), and large (*d* = 0.8) effects (39).

## Results

**Figure 1:**
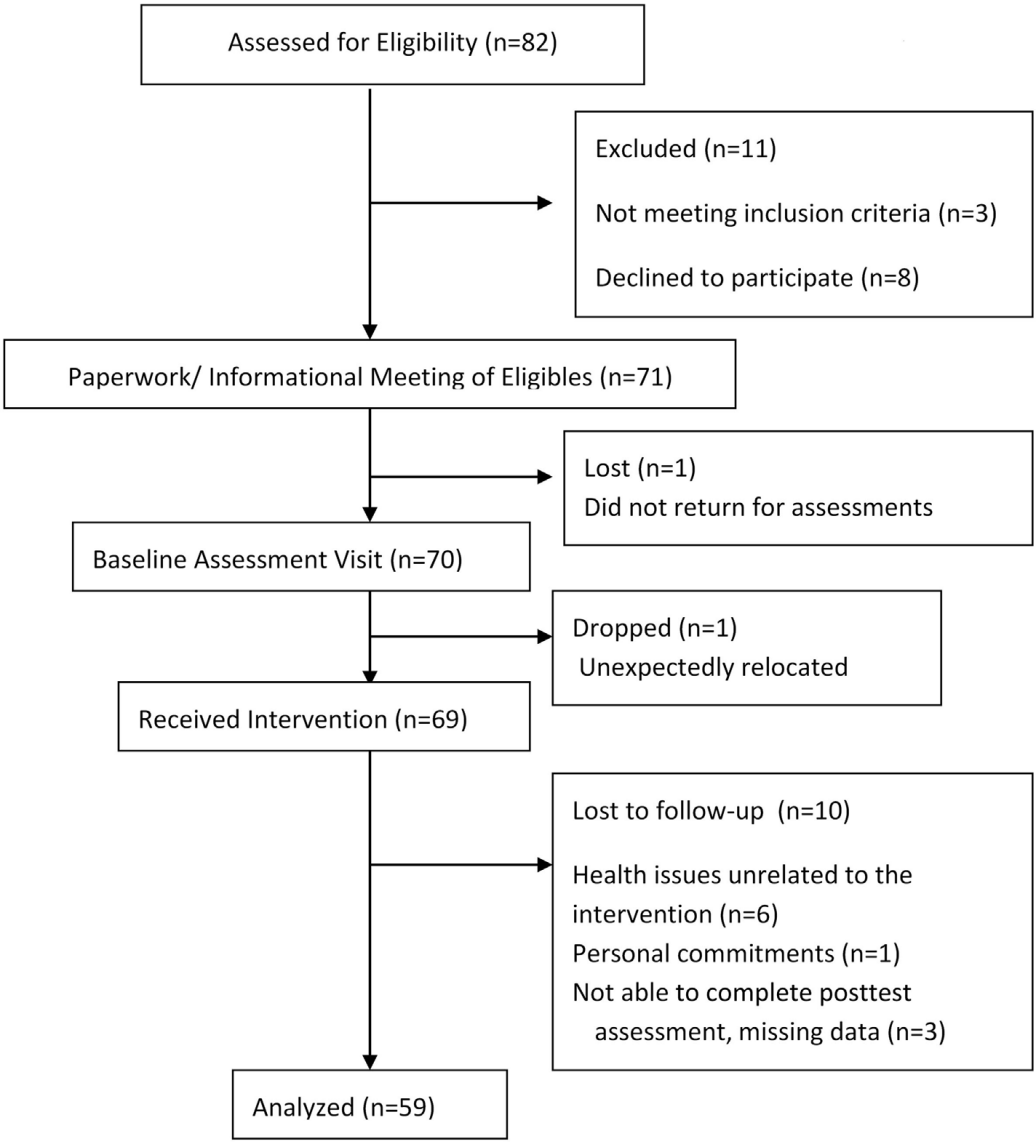
**CONSORT diagram of participant flow**.

Figure 1 provides the CONSORT flow of participants through the program. During recruitment, 111 older adults expressed an interest in the SIB Program, and 82 were screened for participation. Of those screened, eight chose not to participate in the SIB and three did not qualify for the study. A total of 71 participants enrolled in this study; however, one participant did not return for baseline assessments of physical function and one moved prior to the start of the program, resulting in 69 people (60–100 years of age) participating the SIB Program. An additional 10 were lost to follow-up resulting in data on 59 individuals at all time points.

Baseline participant characteristics are presented in Table 1 for all SIB Program participants, and broken down by completers and non-completers. The mean age of the individuals who completed the program was 78.12 ± 6.22 years of age, nearly 76% of the participants were females, and, based on body mass index, 30.43% of participants were classified as obese (BMI ≥ 30.0 kg/m^2^). This sample of older adults reported high falls self-efficacy (FES-I) or low concern about falls (25) despite nearly 85% reporting a fear of falling. The sample was considered underactive (score <6) by their RAPA scores (14). Nearly 46% of our sample reported taking four or more prescription medications, and 47% reported use of an assistive device (e.g., cane or walker) at least some of the time.

**Table 1:** **Baseline characteristics by completion status**.

	Total (*n* = 69)	Completers (*n* = 59)	Non-completers (*n* = 10)	Test statistic	*p* Value
Age, years	78.1 ± 6.66	78.12 ± 6.22	77.80 ± 9.22	0.14	0.89
Female, %	76.81	76.27	80.00	0.07	0.80
Body mass index, kg/m^2^	27.3 (8.9)	28.92 ± 7.30	28.73 ± 7.70	0.08	0.94
Waist circumference, cm	97.72 ± 17.98	98.43 ± 17.15	93.58 ± 22.92	0.79	0.43
Fall in past year, %	46.38	47.46	40.00	0.19	0.66
Fear of falling, %	86.96	84.75	100.00	1.75	0.19
Fall Efficacy Scale International score, 16–64	27.11 ± 8.28	26.84 ± 8.28	28.71 ± 8.54	-0.66	0.51
Rapid Assessment of Physical Activity score, 0–8	3.65 ± 1.73^†^	3.62 ± 1.75	3.80 ± 1.75	-0.30	0.77
Living alone, %	37.31	37.93	33.33	0.07	0.79
Medication (≥4), %	44.93	45.76	40.00	0.11	0.73
Walking aid, %	51.52	47.37	55.56	0.21	0.65
LE osteoarthritis, %	61.19	66.67	30.00	4.82	0.03*
Joint replacement, %	28.99	32.20	10.00	2.05	0.15
Diabetes mellitus, %	30.16	26.42	50.00	2.22	0.14
Hypertension, %	66.18	63.79	80.00	1.00	0.32
Osteoporosis, %	40.00	42.00	30.00	0.50	0.48
Depression, %	15.87	16.67	11.11	0.18	0.67
Timed-up and go, s	8.87 (2.53)	8.78 (2.87)	10.24 (6.22)	1.69	0.09
Berg balance, score	50.00 (6.00)	50.00 (6.00)	49.00 (9.00)	-0.15	0.88
Chair stand, ^#^	9.73 ± 3.39	9.88 ± 3.43	8.90 ± 3.21	0.84	0.40
Arm curl, ^#^	13.11 ± 2.84	13.24 ± 2.87	12.33 ± 2.60	0.89	0.38

*Normal data reported as mean ± SD; non-normal data reported as median (IQR)*.

*p < 0.05.

*^†^n = 68*.

*^#^, Number of repetitions*.

### Attrition

Of the 69 participants who initiated the SIB Program, 10 (14.5%) did not complete the intervention or post-assessments. The main reasons for non-completion were health issues unrelated to the intervention, personal commitments, and not being able to attend the scheduled posttests (Figure 1). Only one difference was found between SIB Program completers and non-completers (completers reported higher rates of lower extremity osteoarthritis than non-completers at baseline; 66.67 versus 30.00%), so further analyses were conducted on completers only.

### Attendance and Falls

Participants’ attendance averaged 21 of the 24 possible meeting days for the 12-week intervention, resulting in an 87.5% attendance rate (range 13–24 days). Of the 40 participants that turned in falls calendars, 9 individuals reported having a fall during the 12-week intervention period, 4 of whom reported multiple falls. None of the reported falls occurred during the exercise portion of the SIB Program.

### Intervention Outcomes

**Figure 2:**
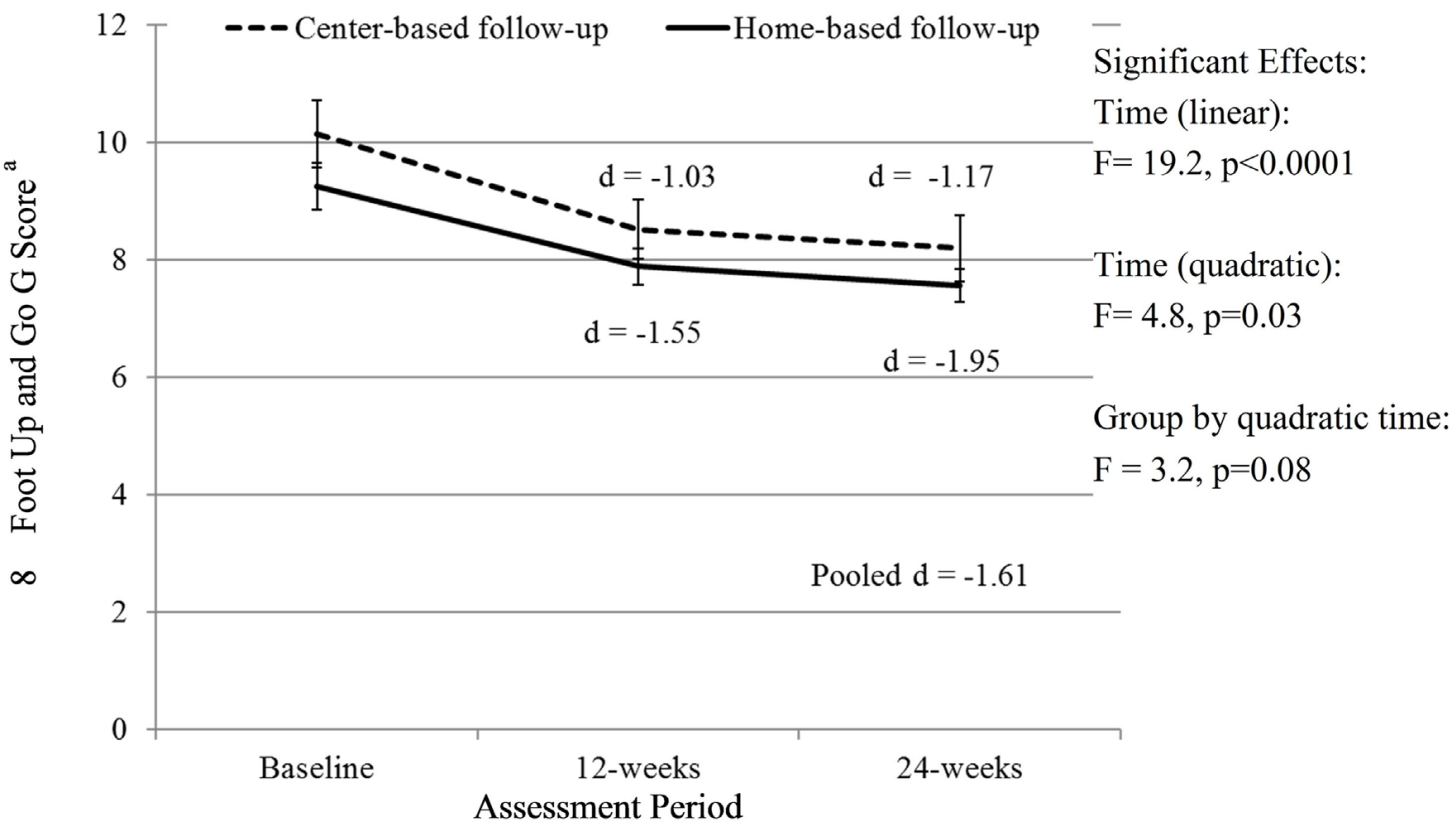
**Change in 8-Foot Up and Go score over time**. Analyses controlled for age, gender, and BMI at baseline and clustered on site membership, and all participants initially received the Stay in Balance Program in a group setting; ^a^a lower score indicates better function.

There was a significant linear (*F* = 19.2, *p* < 0.0001) and quadratic (*F* = 4.8, *p* = 0.03) effect of time and a trend for a group by quadratic time effect (*F* = 3.2, *p* = 0.08) for the 8-Foot Up and Go scores. The quadratic effect of time indicates that the slope of time effects differed from baseline to 12 weeks and from 12 to 24 weeks. Specifically, the improvement in 8-Foot Up and Go scores was greater from baseline to 12 weeks (when all participants received the SIB program) than from 12 to 24 weeks, but there was still a significant improvement from 0 to 24 weeks. There was a trend (*p* = 0.08) for a significant group by quadratic time effect with those attending the center-based exercise program trending toward continual improvement from weeks 12 to 24 (Figure 2).

**Figure 3:**
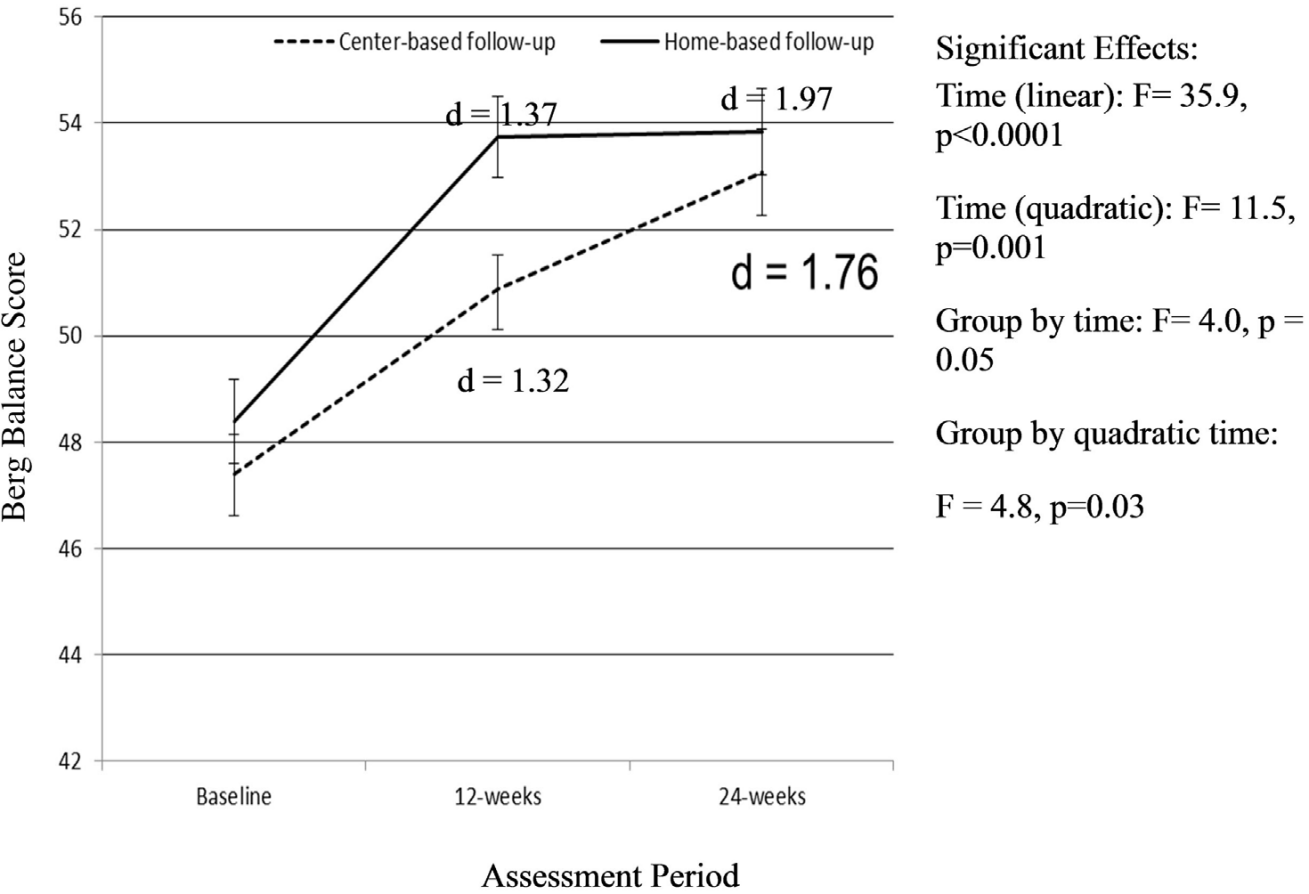
**Change in Berg Balance Score over time**. Analyses controlled for age, gender, and BMI at baseline and clustered on site membership, and all participants initially received the Stay in Balance Program; a higher score indicates better balance.

There was a significant linear effect of time as well as a quadratic effect of time (*F* = 11.5, *p* = 0.001) on the BBS (Figure 3). For the BBS, there was also a group-by-time (*F* = 4.0, *p* = 0.05) and a quadratic time by group (*F* = 4.8, *p* = 0.03) effect on balance. Individuals assigned to the home-based exercise program follow-up group had greater improvements in balance during the initial SIB Program compared to those assigned to the center-based exercise program follow-up group. The improvements in the home-based group were maintained over the 12-week follow-up period. The participants assigned to the follow-up, center-based exercise program did not improve as much as those assigned to the home-based follow-up group during the initial SIB Program. However, this group continued to increase over the 12-week center-based exercise program follow-up period (Figure 3).

**Figure 4:**
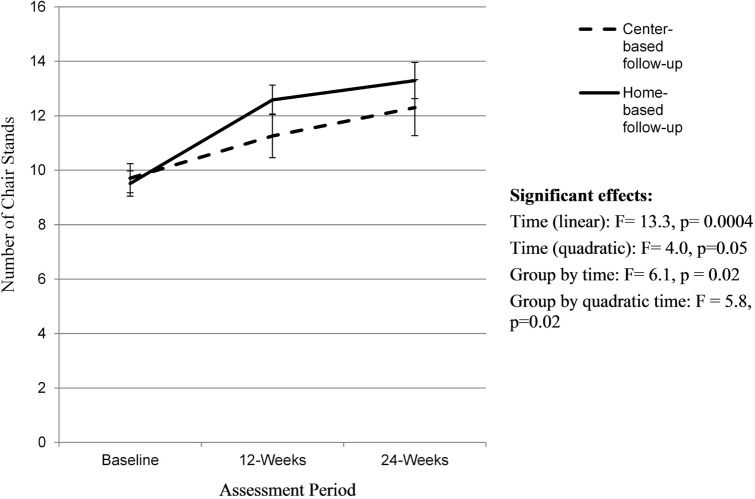
**Changes in 30-s chair stands over time**. Analyses controlled for age, gender, and BMI at baseline and clustered on site membership, and all participants initially received the Stay in Balance Program; a higher score indicates better leg strength.

There was a significant linear (*F* = 13.3, *p* = 0.0004) and quadratic effect of time (*F* = 4.0, *p* = 0.05) on 30-s chair stand scores (Figure 4). There was no effect of group on chair stand results, but there was a group-by-time (*F* = 6.1, *p* = 0.02) and a quadratic time by group effect (*F* = 5.8, *p* = 0.02). Individuals assigned to the home-based follow-up group saw greater improvements in the number of chair stands (ES = 1.54) during the initial SIB Program compared to those in the center-based follow-group (ES = 0.58) and continued to improve over the 12-week follow-up period (ES 0.46). The group assigned to the home-based follow-up saw a greater improvement over time relative to the center-based program (ES 1.5 versus 0.79).

**Figure 5:**
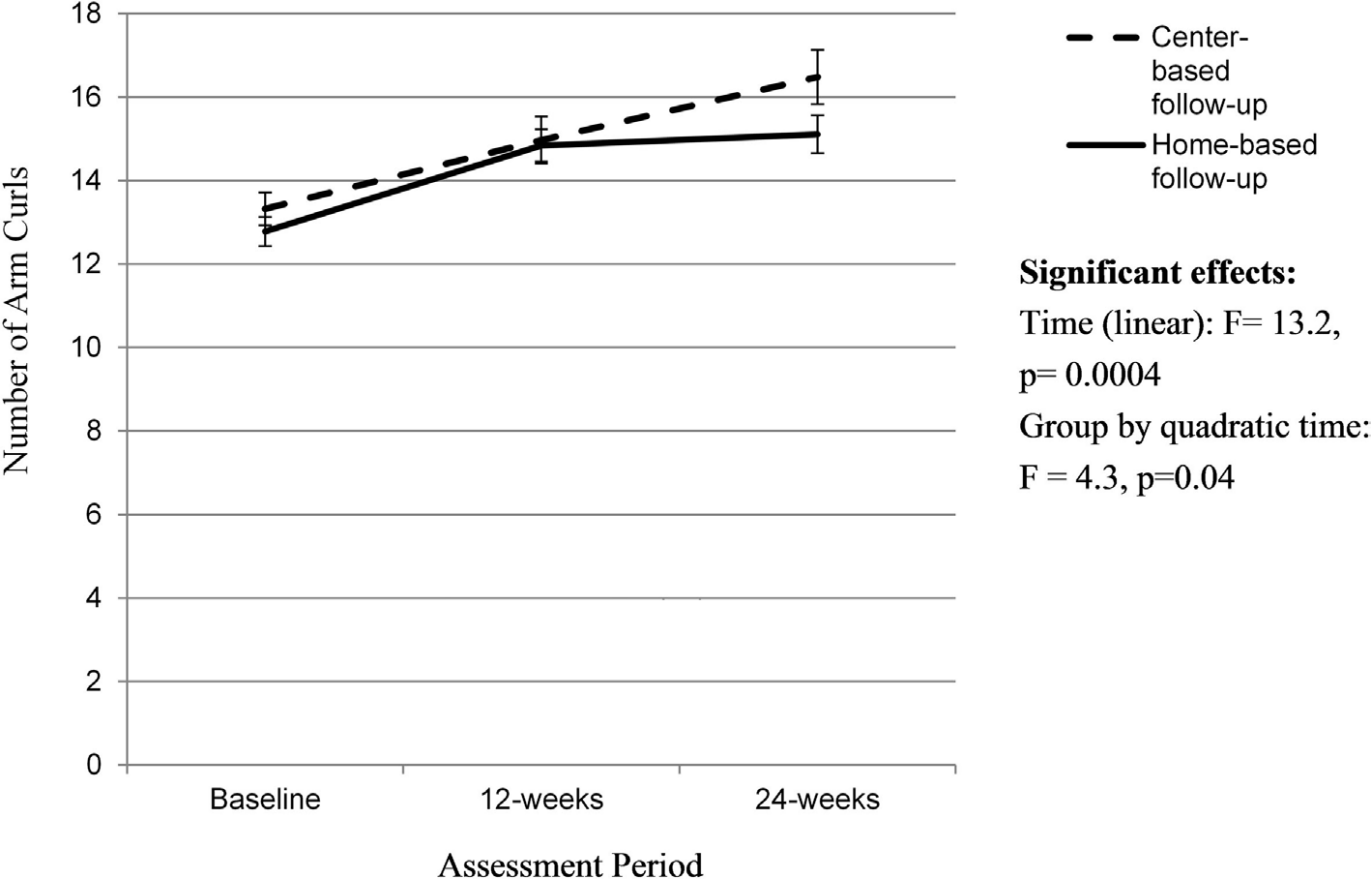
**Change in 30-s arm curls over time**. Analyses controlled for age, gender, and BMI at baseline and clustered on site membership, and all participants initially received the Stay in Balance Program; a higher score indicates better arm strength.

There was a significant linear effect of time (*F* = 13.2, *p* = 0.0004) and a quadratic time by group effect (*F* = 4.3, *p* = 0.04) on number of arm curls (Figure 5). Individuals assigned to the home-based follow-up group saw greater improvements in the number of arm curls (ES of 1.17) during the initial SIB Program compared to those in the center-based follow-group (ES 0.73) and maintained this improvement over the 12-week follow-up period (ES 0.17). The participants assigned to the center-based follow-up did not increase their number of arm curls as much during the initial 12-week SIB Program (ES 0.73) but continued to increase over the 12-week center-based exercise program follow-up period (ES 0.76). However, there were no overall group-by time-effects observed in arm curl results.

## Discussion

Improving balance and lower-extremity strength is necessary for improving falls risk in older adults. The purpose of this intervention was to determine the efficacy of an interdisciplinary, allied health-care student-led, multicomponent FPP (SIB) on balance and physical function in community-dwelling older adults at-risk of falling. A secondary purpose was to compare the effects of assignment to a follow-up, 12-week home-based or center-based exercise program on balance and physical function. Improving balance and lower-extremity strength is necessary for improving falls risk in older adults. Physical performance, functional balance, and leg strength are critical components of fall prevention in older adults (40–42). Findings from this efficacy indicate participation in the multicomponent SIB Program resulted in significant improvements in measures of balance and physical function among older adults at an increased risk of falling. Significant improvements over time were observed for the 8-Foot Up and Go, Berg Balance Scores, 30-s chair stands, and 30-s arm curls after completion of the initial 12-week SIB Program, and these improvements were either maintained or enhanced by allocation to a follow-up center or home-based exercise program.

Exercise is identified as a critical component of FPPs (6, 43). Recent comprehensive reviews and meta-analyses indicate that exercise programs targeting fall prevention need to include at least 50 contact hours over 24 weeks and consist of progressive lower-extremity strengthening exercises and progressive balance exercises (10, 11). Due to limitations in space, time, and staff, this extensive of a program may not be feasible or practical in community-based settings serving older adults. Therefore, a secondary purpose of this study was to compare the effects of a follow-up 12-week home-based or center-based exercise program after completion of the SIB Program. This would result in the suggested 50 contact hours and 24 weeks of exercise participation. For the primary outcome, the 8-Foot Up and Go, the observed improvements were greater from baseline to 12 weeks (when all participants received the SIB Program) than from 12 to 24 weeks, but there was still a significant improvement from 0 to 24 weeks. There was a trend (*p* = 0.08) for only those attending the center-based exercise program toward continual improvement from weeks 12 to 24 (Figure 2). However, this should be interpreted with caution because there may have a ceiling effect in the home-based program; adjusted Up and Go scores for the home-based group were 7.89 after completion of the SIB Program.

Similarly, for Berg Balance Scores, different improvement patterns were observed for the home and center-based groups over time. Participants allocated to the home-based, follow-up exercise program had greater improvements over time during the initial SIB Program, and these improvements were maintained overtime. Conversely, the center-based, follow-up exercise program group had smaller improvements during the SIB Program but continued to improve during the center-based exercise program. It is difficult to determine if the group allocation (home-based versus center) drove the differences or if it was other factors due to the quasi-experimental design of the study. It is plausible the home-based participants likely did not continue to increase during the home-based exercise program due to a ceiling effect. The adjusted mean score on the BBS was 53.74 with an SE of 0.76, and the maximum score on the BBS is 56. Additionally, the participants on average saw a 6-point improvement in Berg Balance, and it has been suggested that a 4-point improvement in the BERG scale is suggestive of improved functional balance for individuals with a starting Berg score between 45 and 56 (36).

Lower extremity strength as assessed by 30-s chair stands increased over time for both groups with no effect of group. Similar to the findings on the BBS, individuals assigned to the home-based follow-up group saw significantly greater improvements in the number of chair stands during the initial SIB Program compared to individuals allocated to the center-based follow-up group and continued to improve over the 12-week follow-up period. The participants assigned to the center-based follow-up did not increase their number of chair stands as much during the initial 12-week SIB Program but still continued to increase over the 12-week center-based exercise program follow-up period. Contrary to our hypotheses, the group assigned to the home-based follow-up saw a greater improvement over time relative to the center-based program, and this discrepancy was primarily due to gains during the initial SIB Program. Similarly, there was a quadratic group-by-time effect for arm strength. A similar pattern emerged for the home versus center-based follow-up groups. The group allocated to the follow-up home-based exercise program saw greater improvements in arm curls during the initial SIB Program and maintained these improvements over time. The group allocated to the center-based, follow-up exercise program saw smaller improvements during the SIB Program but continued to improve. Unlike lower extremity strength, there was no overall group-by-time effect.

The current sample of older adults scored lower than their age matched norms on multiple physical assessment measures. Their TUG time was slower than norms (4.2–7.1 s) (28). Furthermore, the chair stand and arm curl scores were also below their matched norms (chair stands = 10–17, arm curls = 12–21) (28). This sample of community-dwelling older adults was classified as low fall risk according the BBS (scores 45–56) (31). Although the BBS score classified participants in this intervention as low risk of falling, significant findings were still found in all physical assessment outcome measures. This significance is most likely due to the strategic recruitment of participants that were classified as “at-risk” of falling based on previously established risk factors for falling (history of falls, age ≥80 years, female gender, ≥4 medications, presence of osteoarthritis in the lower extremity or expressed a fear of falling). The BBS may not be able to detect subtle balance impairments that are predictive of falls in ambulatory, community-dwelling individuals due to the ceiling effect observed in this population. However, we were still able to observe improvements in scores suggesting that the intervention did improve balance in individuals at low risk for falls or for whom the BBS could not detect balance impairments. Future research should utilize more precise evaluations of balance including center of pressure assessments using a force plate or inertial movement unit.

Collectively, these findings suggest there is a need for an initial group-based FPP that emphasizes physical activity to improve balance and physical function outcomes. The effects of the program can be maintained through home exercise. Offering a continual group-based exercise program may confer greater benefits than asking individuals to exercise on their own. Based on the observed results, it is also plausible that individuals initially assigned to continue the exercises at home versus as part of a group may be more motivated to work harder during the program. In this study, we did not observe any effects of group for any of the outcome variables, but we did observe linear group-by-time and/or quadratic group-by-time interactions. The majority suggested the group assigned to the follow-up home-based group improved more during the initial SIB Program and maintained the outcomes. In contrast, those assigned to the center-based follow-up group improved to a lesser extent during the initial site-based SIB Program but continued to improve during the center-based exercise program. These findings could potentially be explained by compensatory rivalry; these participants knew ahead of time which follow-up group they were assigned to. Future evaluations should focus on random assignment to home or center-based follow-up exercise groups after the initial SIB Program.

### Strengths and Limitations

The primary limitation of this study was the quasi-experimental design. All participants received the initial SIB Program, and participants were allocated to either a group-based or home-based exercise program based on site preference. There was no control or comparison group so we cannot say the intervention caused the outcomes. However, the study participants were sedentary individuals with an elevated risk for falling. It is highly unlikely that the assessed outcomes would have improved in the absence of a physical activity intervention. The normal trajectory for physical function and balance with aging is a decline. The participants’ knowledge of follow-up group assignment may have reduced the internal validity of the study though. The participants assigned to the home-based program had greater gains during the SIB Program for the majority of the outcomes, and this could be indicative of compensatory rivalry. The study used well-established objective measures of physical function and balance outcomes enhancing the validity of findings.

### Summary and Conclusion

Study results suggest the SIB Program is an effective program for improving balance and physical function outcomes in older adults who are at-risk for falling. The program can be delivered by allied health-care students potentially enhancing the sustainability of the program and reducing program costs. More research needs to be done to evaluate the effects of allocating individuals to a home-based exercise program versus a center-based exercise program after the completion of a multicomponent exercise programs. The effects of group assignment in the present study were mixed. For the 8-Foot Up and Go and arm curls, there was no linear group-by-time effect; however, there was a quadratic time by group effect, which suggested participants who knew they were going to have to exercise on their own after the program finished had greater gains during the program on these outcomes. These individuals maintained their improvements through home-based exercises whereas those assigned to the center-based exercise program continued to improve. For the BBS and the 30-s chair stand, there was a linear effect of time that favored the group allocated to the home-based follow-up program. This effect was primarily driven by larger gains during the initial SIB Program. More research is warranted to investigate the optimal ways to sustain improvements in balance and physical function outcomes over time after completion of a multicomponent FPP.

## Author Contributions

CD contributed to the design and implementation of the study, co-developed the Stay in Balance Program, assisted with data collection and data analysis, and wrote the draft of the manuscript. MM contributed to the design and implementation of the study, co-developed the Stay in Balance Program, was responsible for all data collection and management, assisted with data analysis, and provided critical feedback on the manuscript draft. MB’s primary role was data analysis and interpretation. He also assisted with the writing of the manuscript and provided critical, editorial feedback on the final manuscript.

## Conflict of Interest Statement

The authors of this study declare that this research was conducted in the absence of any commercial or financial relationships that could be construed as a potential conflict of interest.
